# Supplementary material for: Cebp1 and Cebpβ transcriptional axis controls eosinophilopoiesis in zebrafish
Source: Nat Commun. 2024 Jan 27;15:811. doi: 10.1038/s41467-024-45029-0 (PMC10821951; doi:10.1038/s41467-024-45029-0)

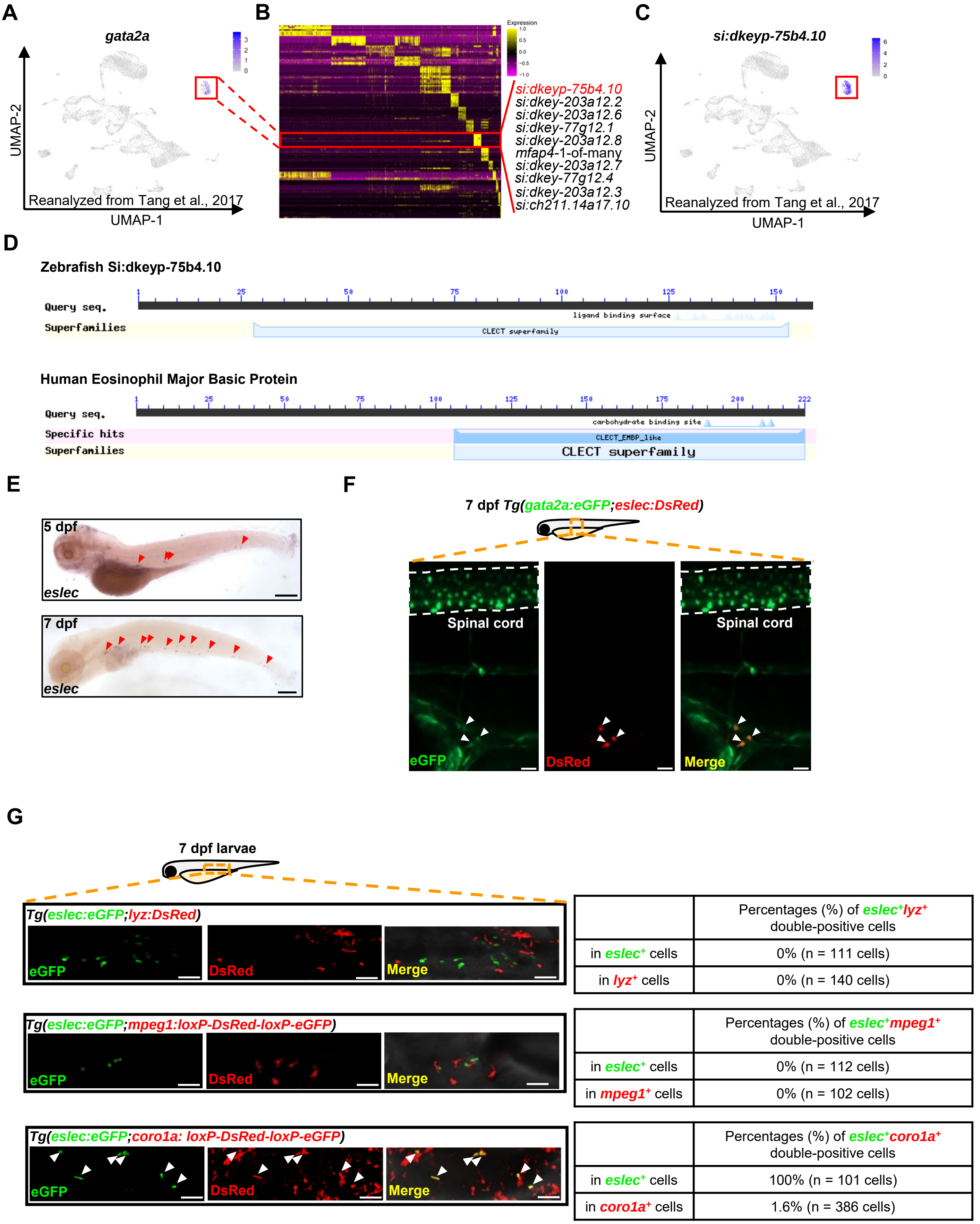

	Percentages (%) of *eslec*⁺*lyz*⁺ double-positive cells
in *eslec*⁺ cells	0% (n = 111 cells)
in *lyz*⁺ cells	0% (n = 140 cells)

	Percentages (%) of *eslec*⁺*mpeg1*⁺ double-positive cells
in *eslec*⁺ cells	0% (n = 112 cells)
in *mpeg1*⁺ cells	0% (n = 102 cells)

	Percentages (%) of *eslec*⁺*coro1a*⁺ double-positive cells
in *eslec*⁺ cells	100% (n = 101 cells)
in *coro1a*⁺ cells	1.6% (n = 386 cells)

Figure S1. Mining eosinophil marker *eslec* from scRNA-Seq data. (A) UMAP showing the expression pattern of *gata2a*. The dataset of Tang et al. 2017 was reanalyzed and the expression pattern of *gata2a* was shown with UMAP. (B) Top 10 markers of the *gata2a*⁺ cells. (C) UMAP showing the expression pattern of *si:dkeyp-75b4.10*. (D) Protein domain of Si:dkeyp-75b4.10 (Eslec) and human Eosinophil Major Basic Protein (obtained from NCBI). Blue boxed regions indicate the CLECT superfamily domain. (E) Expression pattern of *eslec* in zebrafish larvae. WISH exhibited that *eslec*⁺ cells (red arrowheads) could be found since 5 dpf and increased at 7 dpf (bar = 200 μm, red box showing enlarged view). (F) Colocalization of *eslec*:DsRed and *gata2a*:eGFP signals. The DsRed⁺ cells were all eGFP^{low} (white arrowheads), while eGFP⁺DsRed⁻ cells were abundantly found in the spinal cord (white dotted line-surrounded) and other tissue (bar = 20 μm). (G) Colocalization of *eslec*⁺ cells with other myelocytes. The *eslec*⁺ cells showed no colocalization with *lyz*⁺ or *mpeg1*⁺ cells, while they are all colocalized with *coro1a*⁺ cells (white arrowhead, bar = 50 μm). The right table showed the quantification results. In *Tg(eslec:eGFP;lyz:DsRed)* and *Tg(eslec:eGFP;mpeg1:loxP-DsRed-loxP-eGFP)* larvae, no *eslec*⁺ cell was DsRed⁺, and no *lyz*⁺ or *mpeg1*⁺ cell was eGFP⁺. In *Tg(eslec:eGFP;coro1a:loxP-DsRed-loxP-eGFP)* larvae, all *eslec*⁺ cells were DsRed⁺, while only 1.6% of *coro1a*⁺ cells were eGFP⁺.

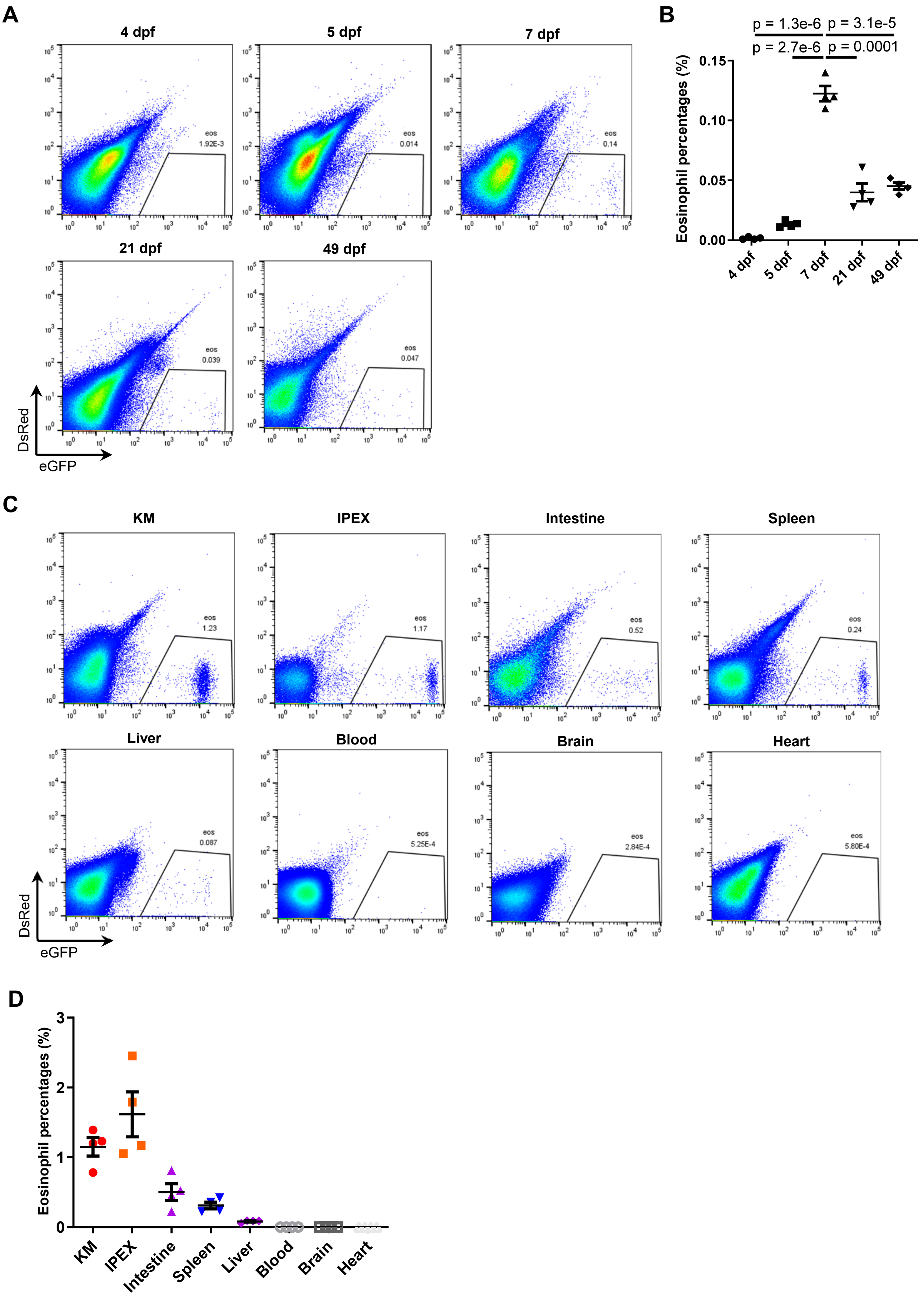

Figure S2. Flow cytometry showing the eosinophil pattern. (A) Flow cytometry showing the eosinophil percentages from 4 dpf to 49 dpf zebrafish. Eosinophils were gated on eGFP^{+}DsRed^{-} cells to exclude the auto-fluorescent cells. (B) Quantification of (A). All other groups were only compared with the 7 dpf group. (Student's *t*-test, two-sided, mean ± SEM). (C) Flow cytometry showing the eosinophil percentages in different adult *Tg(eslec:eGFP)* zebrafish tissues. Eosinophils were gated on eGFP^{+}DsRed^{-} cells to exclude the auto-fluorescent cells. (D) Quantification of (C). Two independent experiments were conducted with n = 4 in each group. Source data are provided as a Source Data file.

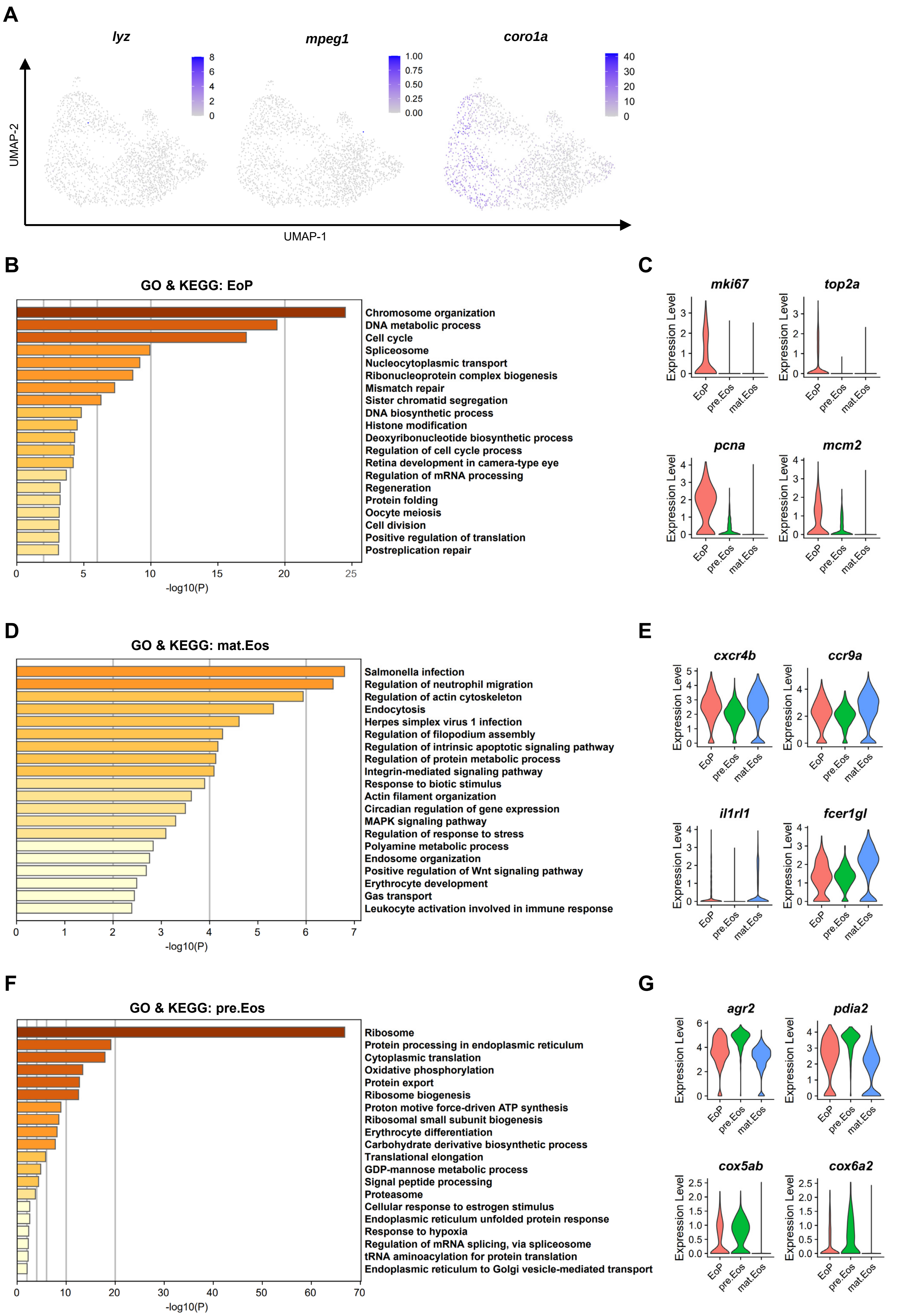

Figure S3. The features of EoP, pre.Eos, and mat.Eos. (A) The expression pattern of *lyz*, *mpeg1*, and *coro1a* in eosinophils. (B) The GO/KEGG annotations of EoP marker genes. (C) Violin plots showing the expression patterns of featured EoP marker genes. (D) The GO/KEGG annotations of mat.Eos marker genes. (E) Violin plots showing the expression patterns of featured mat.Eos marker genes. (F) The GO/KEGG annotations of pre.Eos marker genes. (G) Violin plots showing the expression patterns of featured pre.Eos marker genes.

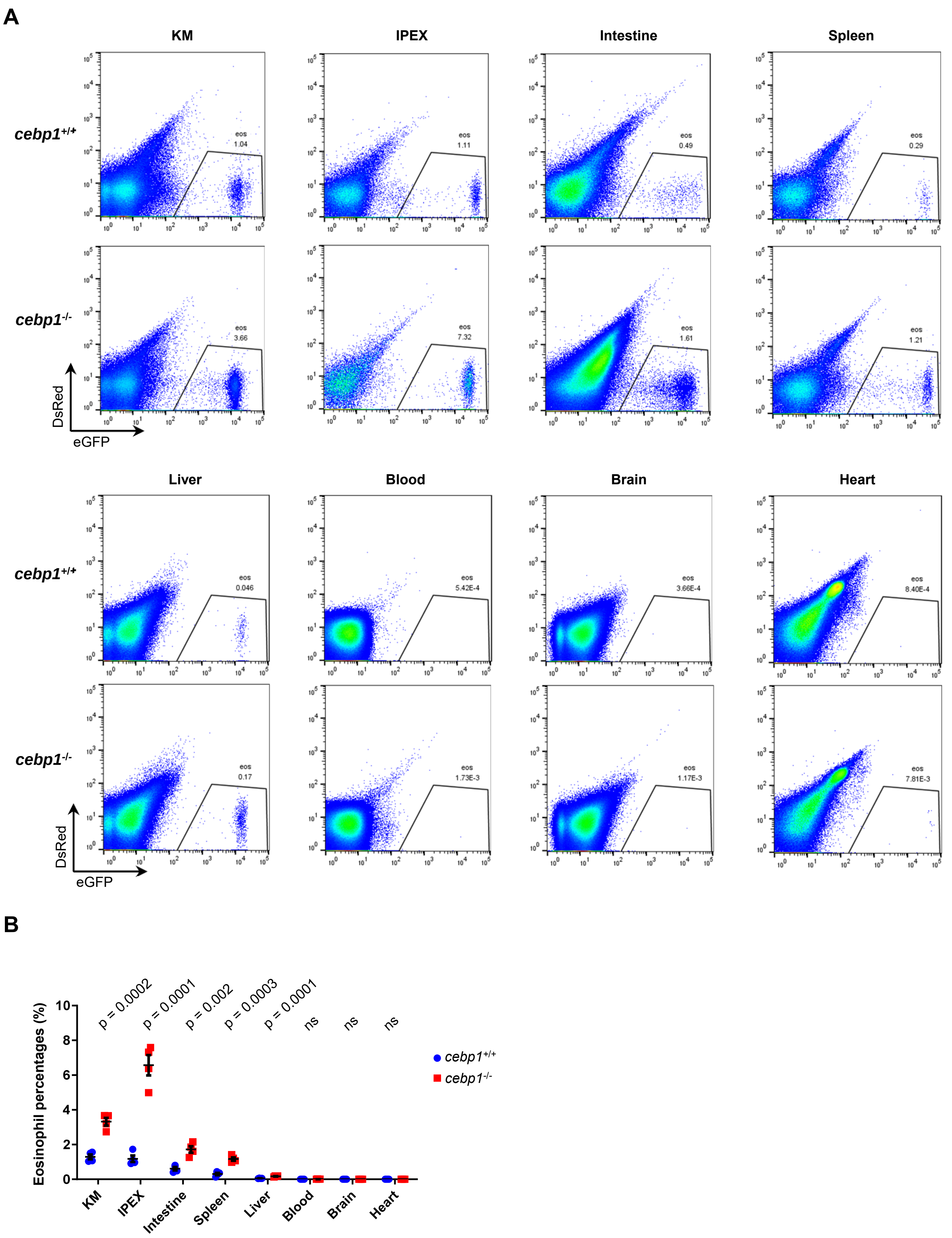

Figure S4. Eosinophil distributions in *cebp1* mutants. (A). The eosinophil percentages in different tissues of *cebp1*$^{+/+}$ and *cebp1*$^{-/-}$ *Tg(eslec:eGFP)* zebrafish. Eosinophils were gated on eGFP^{+}DsRed^{-} cells to exclude the auto-fluorescent cells. (B) Quantification of (A). (Student's *t*-test, two-sided, mean ± SEM). Two independent experiments were conducted with n = 4 in each group. Source data are provided as a Source Data file.

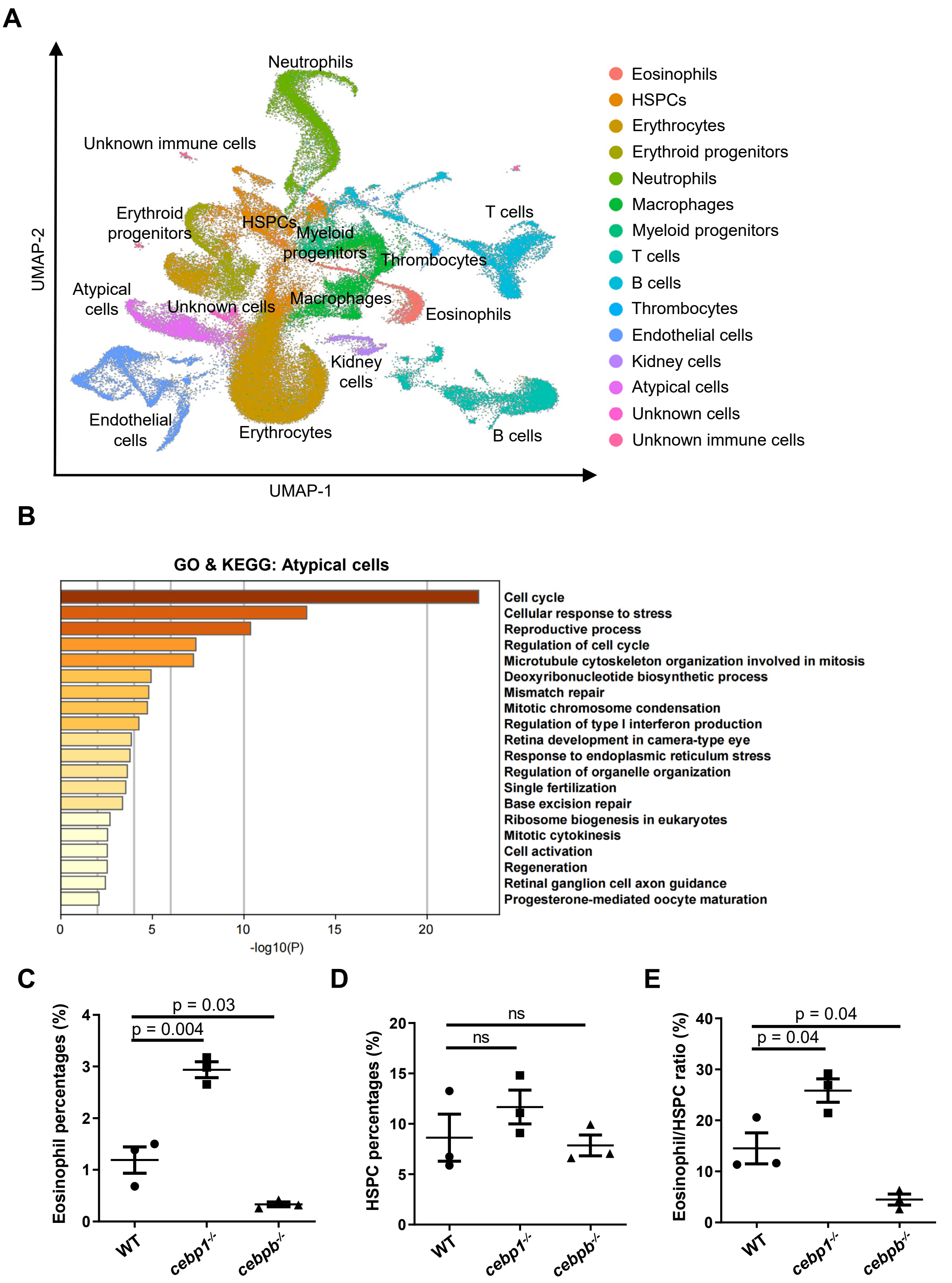

Figure S5. Supporting information for scRNA-Seq data of KM cells from different genotypes. (A) UMAP showing the clustering information of KM cells in the integrated data of WT, *cebp1*$^{-/-}$, and *cebpb*$^{-/-}$ fish. (B) The GO/KEGG annotations of marker genes of the atypical cells. (C) The eosinophil percentages in all KM cells among different genotypes (n = 3, Student's *t*-test, two-sided, mean ± SEM). (D) The HSPC percentages in all KM cells among different genotypes (n = 3, Student's *t*-test, two-sided, mean ± SEM). (E) The eosinophil/HSPC ratios among different genotypes (n = 3, Student's *t*-test, two-sided, mean ± SEM). Source data are provided as a Source Data file.

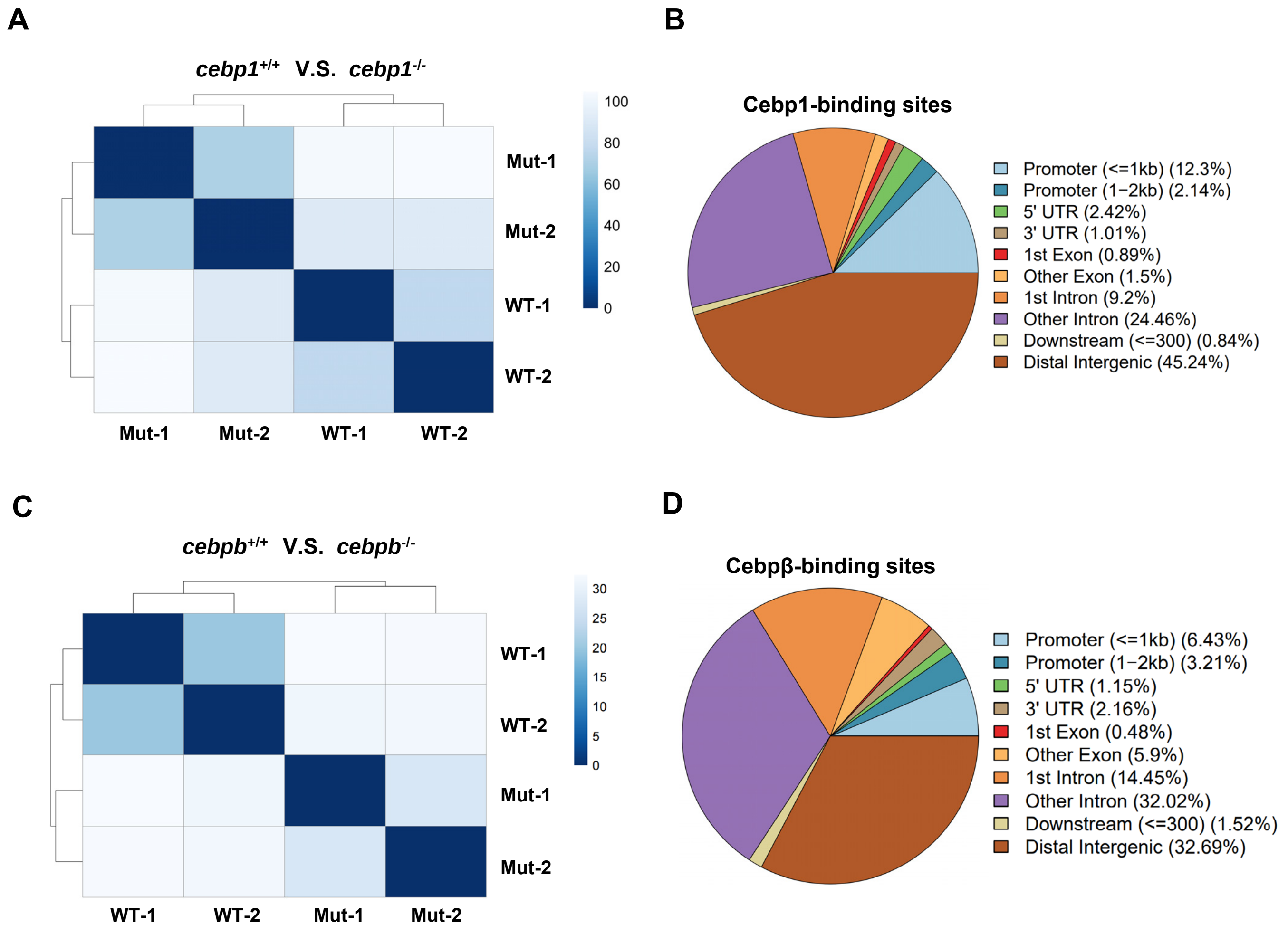

Figure S6. The quality control for sequencing samples. (A) Sample-to-sample heatmap of *cebp1*$^{+/+}$ and *cebp1*$^{-/-}$ eosinophils. The darker color in the heatmap indicates that the samples are more similar. (B) Annotation pie of the locations of Cebp1-binding sites. (C) Sample-to-sample heatmap of *cebpb*$^{+/+}$ and *cebpb*$^{-/-}$ eosinophils. The darker color in the heatmap indicates that the samples are more similar. (D) Annotation pie of the locations of Cebpβ-binding sites.

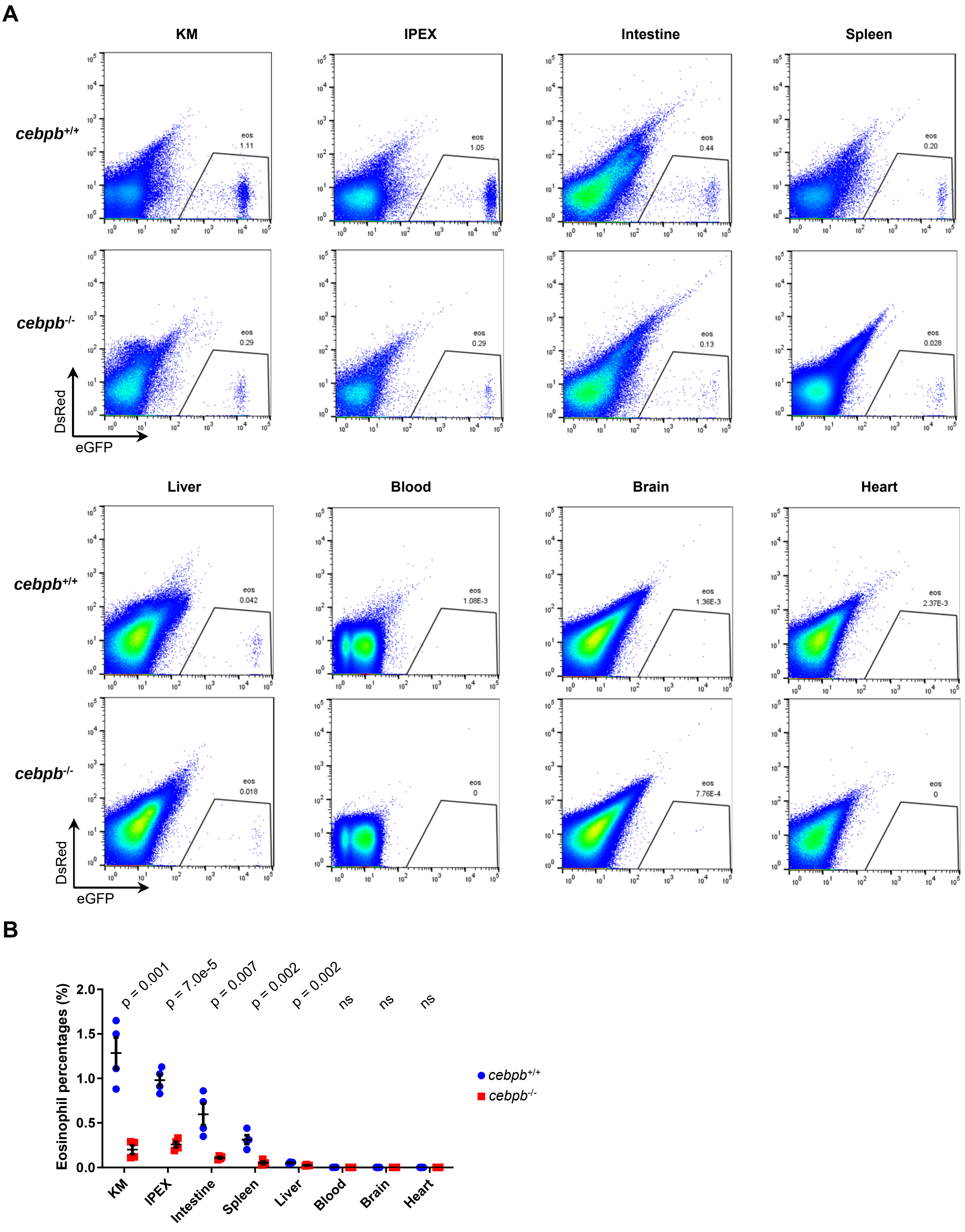

Figure S7. Eosinophil distributions in *cebpb* mutants. (A). The eosinophil percentages in different tissues of *cebpb*$^{+/+}$ and *cebpb*$^{-/-}$ *Tg(eslec:eGFP)* zebrafish. Eosinophils were gated on eGFP^{+}DsRed^{-} cells to exclude the auto-fluorescent cells. (B) Quantification of (A). (Student's *t*-test, two-sided, mean ± SEM). Two independent experiments were conducted with n = 4 in each group. Source data are provided as a Source Data file.

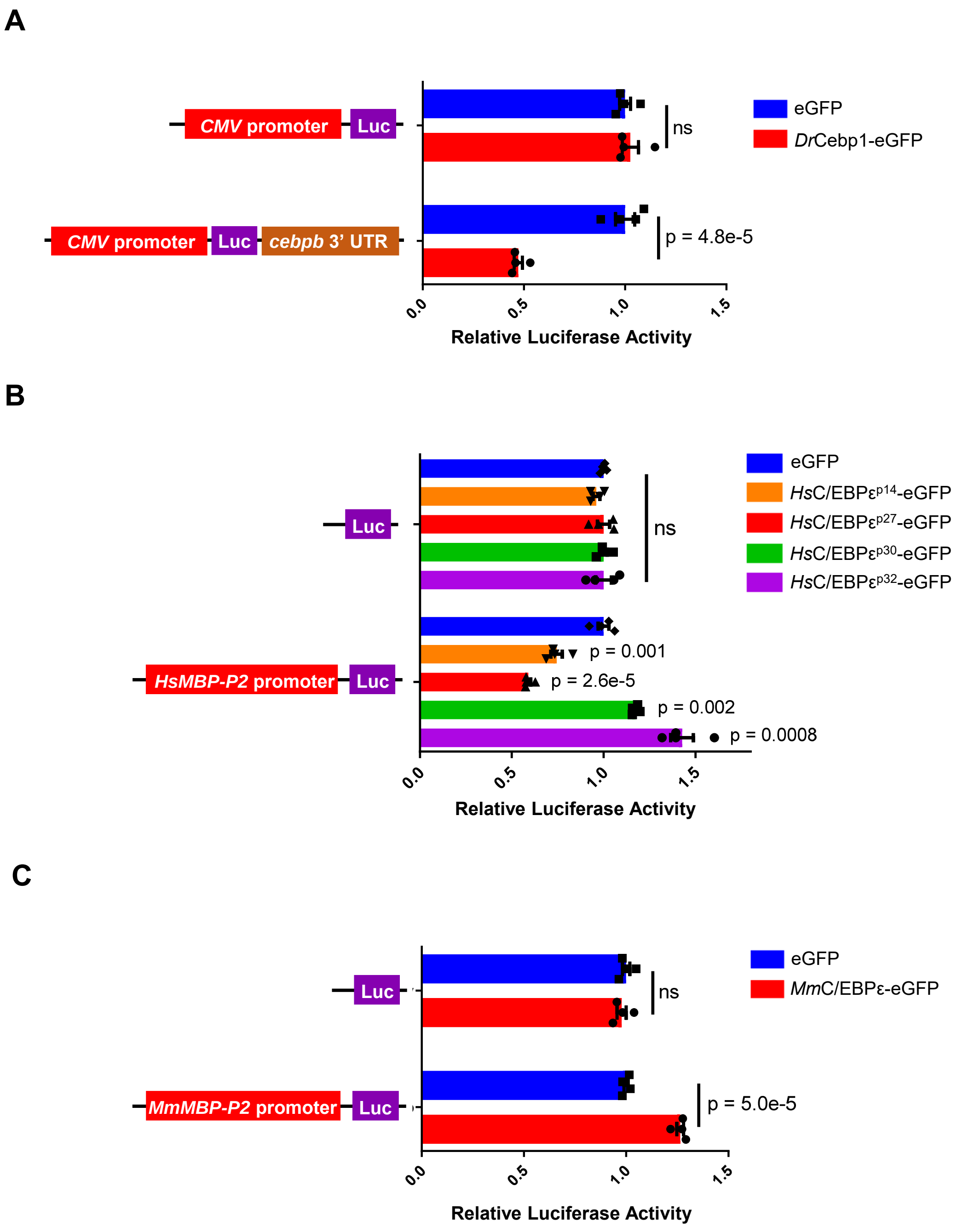

Figure S8. Positive control of the C/EBPε-eGFP fusion proteins. (A) *Dr*Cebp1-eGFP inhibiting the expression of *cebpb*. Luciferase assay demonstrated that *Dr*Cebp1-eGFP inhibited the luciferase activities when the 3' UTR of *cebpb* was added after the luciferase gene (Luc). (B) *Hs*C/EBPε-eGFP affecting the activity of *HsMBP-P2* promoter. Luciferase assay demonstrated that *Hs*C/EBPε^{p14}-eGFP and *Hs*C/EBPε^{p27}-eGFP inhibited the activity of *HsMBP-P2* promoter, while *Hs*C/EBPε^{p30}-eGFP and *Hs*C/EBPε^{p32}-eGFP exhibited activator effects. (C) *Mm*C/EBPε-eGFP inducing the activity of *MmMBP-P2* promoter. Luciferase assay demonstrated that *Mm*C/EBPε-eGFP could significantly enhance the expression of Luc when *MmMBP-P2* promoter was used to trigger Luc expression. All groups were only compared with the eGFP control group of each assay (Student's t-test, two-sided, mean ± SEM, ns represents no significance). Three independent experiments were conducted with n = 4 in each group. Source data are provided as a Source Data file.

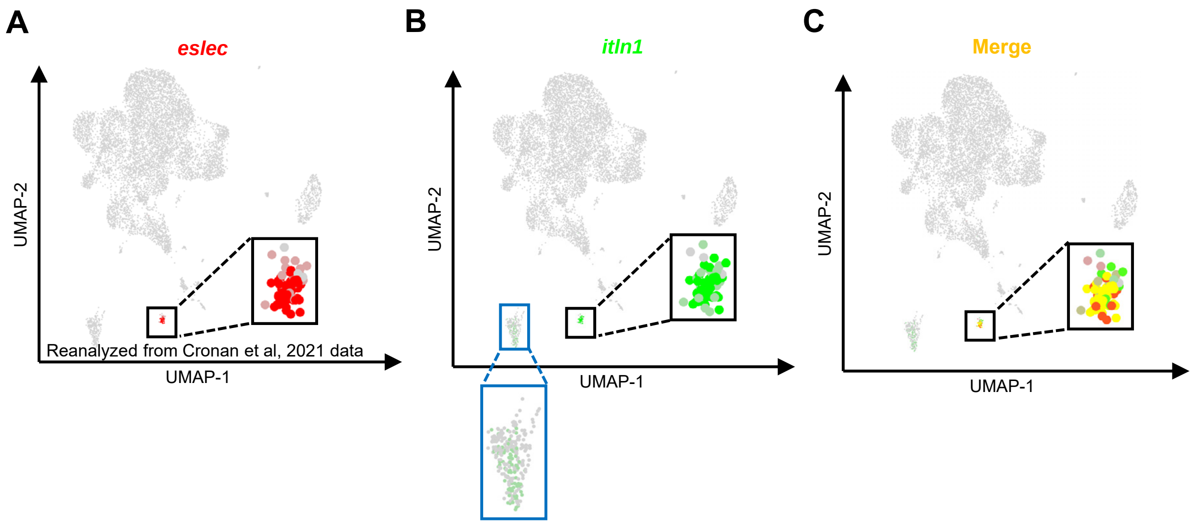

Figure S9. *In silico* verification of *eslec* as an eosinophil marker. Expression of *eslec* (red) and *itln1* (green) in granuloma cells (reanalyzed from Cronan et al, 2021). The black boxes showed the larger views of the predicted eosinophils and the blue box showed the larger view of the predicted neutrophils.

Supplementary information: proportions of cell types in scRNA-Seq data

1. scRNA-Seq of eGFP+ cells from *Tg(eslec:eGFP)* KM

Clusters	Percentages (%)
EoP	21.51
pre.Eos	23.61
mat.Eos	54.88

2. scRNA-Seq of whole KM cells from WT, *cebp1*$^{-/-}$ and *cebpb*$^{-/-}$ fish.

Percentages (%)	WT	*cebp1*$^{-/-}$	*cebpb*$^{-/-}$
Eosinophils	1.14	2.93	0.32
HSPCs	8.00	11.52	8.18
Erythrocytes	33.92	31.13	31.09
Erythroid progenitors	4.64	6.61	3.78
Neutrophils	17.25	0.96	11.15
Macrophages	5.92	7.45	11.16
Myeloid progenitors	2.07	3.42	1.71
T lymphocytes	10.18	5.78	9.08
B lymphocytes	7.81	8.32	9.38
Thrombocytes	0.88	1.14	0.53
Endothelial cells	5.88	5.84	9.91
Kidney cells	0.53	0.86	1.98
Atypical cell	0.26	12.32	0.72
Unknown cells	1.10	0.95	0.65
Unknown immune cells	0.43	0.78	0.37

Supplementary information: gating strategies in flow cytometry

1. Gating strategy of eGFP+ cells from *Tg(eslec:eGFP)* zebrafish

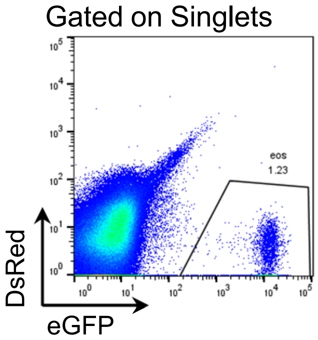

2. Gating strategy of mouse bone marrow cells

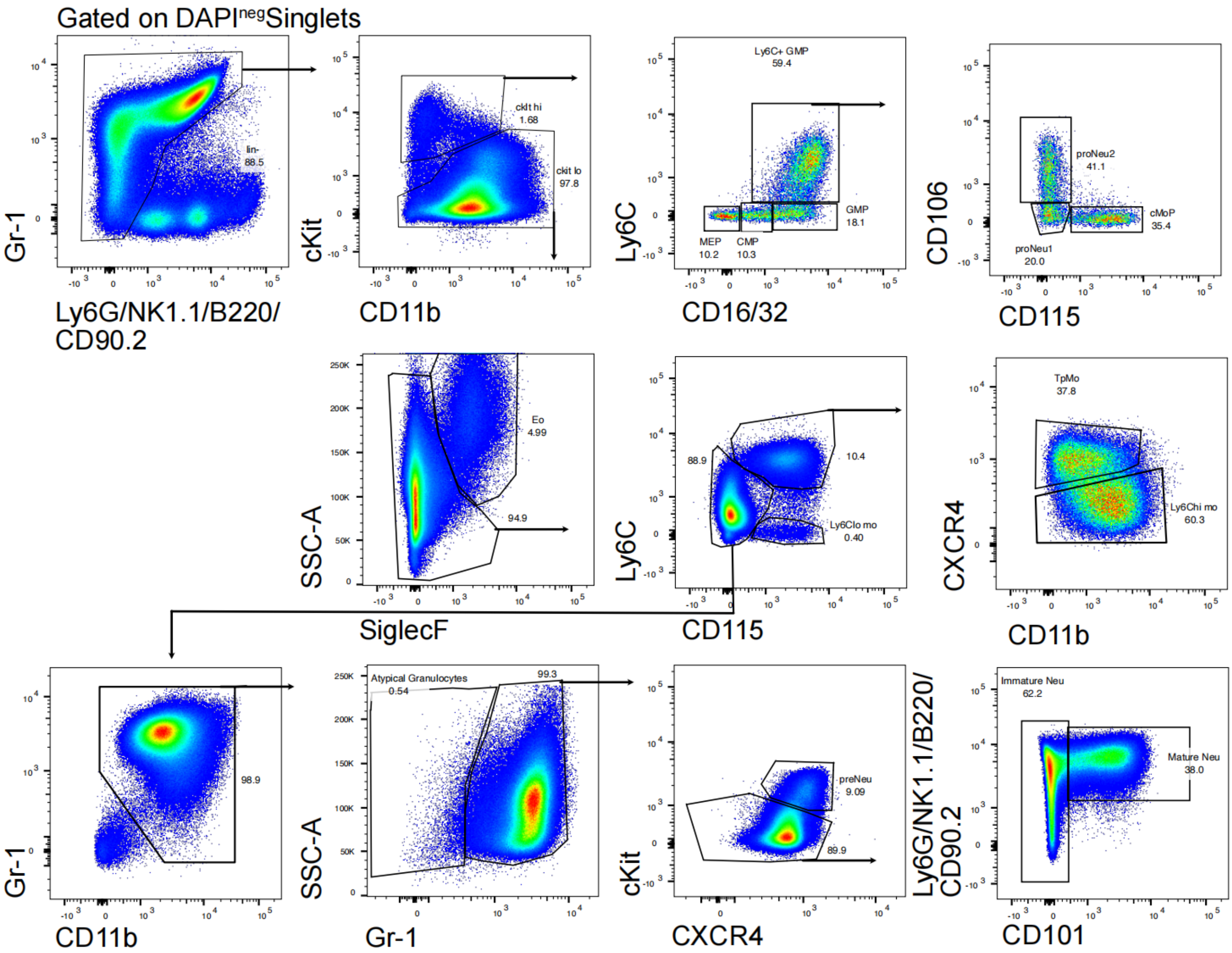

Supplement: Supplementary file 1 — Supplementary information [file 41467_2024_45029_MOESM1_ESM.pdf]
