# Supplementary material for: Cebp1 and Cebpβ transcriptional axis controls eosinophilopoiesis in zebrafish
Source: Nat Commun. 2024 Jan 27;15:811. doi: 10.1038/s41467-024-45029-0 (PMC10821951; doi:10.1038/s41467-024-45029-0)
Supplement: Supplementary file 3 — Description of Additional Supplementary Files [file 41467_2024_45029_MOESM3_ESM.pdf]

Description of Additional Supplementary Files

File Name: Supplementary Data 1
Description: **The marker genes of each eosinophil lineage cell cluster.** The eosinophils ($eGFP^+$) were sorted from adult *Tg(eslec:eGFP)* kidney marrow cells and applied to single-cell RNA-Seq. The highly expressed genes of each cluster (EoP, pre.Eos, and mat.Eos) were shown, along with their p values, fold changes, and the percentages of cells in which they were expressed. The analysis is one-sided.

File Name: Supplementary Data 2
Description: **The gene expression profiles in *cebp1*$^{-/-}$ eosinophils compared with *cebp1*$^{+/+}$ eosinophils.** The eosinophils ($eGFP^+$) were respectively sorted from *cebp1*$^{+/+}$ or *cebp1*$^{-/-}$ *Tg(eslec:eGFP)* larvae and applied to bulk RNA-Seq. The mean expression level, fold change, and p values were shown for each gene. The analysis is two-sided.

File Name: Supplementary Data 3
Description: **The peak profiles of Cebp1 binding to genomic DNA.** The whole larvae of *Tg(hsp70:cebp1-eGFP)* were applied to ChIP-Seq assay, and the input samples served as controls. The peaks were filtered by q value (< 0.05) and distance to the transcription start site (< 2kb). The location, width, signal value, q value, annotation, and distance to the transcription start site were shown for each peak.

File Name: Supplementary Data 4
Description: **The gene expression profiles in *cebpb*$^{-/-}$ eosinophils compared with *cebpb*$^{+/+}$ eosinophils.** The eosinophils ($eGFP^+$) were respectively sorted from *cebpb*$^{+/+}$ or *cebpb*$^{-/-}$ *Tg(eslec:eGFP)* larvae and applied to bulk RNA-Seq. The mean expression levels, fold changes, and p values were shown for each gene. The analysis is two-sided.

File Name: Supplementary Data 5
Description: **The peak profiles of Cebpβ binding to genomic DNA.** The whole larvae of *Tg(hsp70:cebpb-eGFP)* were applied to ChIP-Seq assay, and the input samples served as controls. The peaks were filtered by q value (< 0.05) and distance to the transcription start site (< 2kb). The location, width, signal value, q value, annotation, and distance to the transcription start site were shown for each peak.

File Name: Supplementary Data 6
Description: **The marker genes and differentially expressed genes of each cell type.** The whole kidney cells of adult WT, *cebp1*$^{-/-}$, and *cebpb*$^{-/-}$ fish were applied to single-cell RNA-Seq. Sheet 1: The abbreviations for cell types. Sheet 2: The marker genes of each cell type were shown. The analysis is one-sided. Sheet 3: The differentially expressed genes of each cell type between WT and *cebp1* mutants were shown. The analysis is two-sided. Sheet 4: The differentially expressed genes of each cell type between WT and *cebpb* mutants were shown. The p values, fold changes, and the percentages of cells in which they were expressed were shown for each gene. The analysis is two-sided.

File Name: Supplementary Data 7

Description: **The marker genes and differentially expressed genes of each subcluster of the eosinophil lineage.** The whole kidney cells of adult WT, *cebp1*$^{-/-}$, and *cebpb*$^{-/-}$ fish were applied to single-cell RNA-Seq, and the eosinophils were subsequently subclustered into Eb-P, EoP, pre.Eos, and mat.Eos. Sheet 1: The marker genes of each subcluster were shown. The analysis is one-sided. Sheet 2: The differentially expressed genes of each subcluster between WT and *cebp1* mutants were shown. The analysis is two-sided. Sheet 3: The differentially expressed genes of each subcluster (except for Eb-P since *cebpb* mutants had no Eb-P) between WT and *cebpb* mutants were shown. The p values, fold changes, and the percentages of cells in which they were expressed were shown for each gene. The analysis is two-sided.
