# Supplementary material for: Cebp1 and Cebpβ transcriptional axis controls eosinophilopoiesis in zebrafish
Source: Nat Commun. 2024 Jan 27;15:811. doi: 10.1038/s41467-024-45029-0 (PMC10821951; doi:10.1038/s41467-024-45029-0)
Supplement: Supplementary file 11 — Reporting Summary [file 41467_2024_45029_MOESM11_ESM.pdf]

nature portfolio

Corresponding author(s): Yiyue Zhang

Last updated by author(s): Dec 17, 2023

Reporting Summary

Nature Portfolio wishes to improve the reproducibility of the work that we publish. This form provides structure for consistency and transparency in reporting. For further information on Nature Portfolio policies, see our Editorial Policies and the Editorial Policy Checklist.

Statistics

For all statistical analyses, confirm that the following items are present in the figure legend, table legend, main text, or Methods section.

n/a	Confirmed	
☐	☒	The exact sample size (*n*) for each experimental group/condition, given as a discrete number and unit of measurement
☐	☒	A statement on whether measurements were taken from distinct samples or whether the same sample was measured repeatedly
☐	☒	The statistical test(s) used AND whether they are one- or two-sided<br>*Only common tests should be described solely by name; describe more complex techniques in the Methods section.*
☐	☒	A description of all covariates tested
☐	☒	A description of any assumptions or corrections, such as tests of normality and adjustment for multiple comparisons
☐	☒	A full description of the statistical parameters including central tendency (e.g. means) or other basic estimates (e.g. regression coefficient) AND variation (e.g. standard deviation) or associated estimates of uncertainty (e.g. confidence intervals)
☐	☒	For null hypothesis testing, the test statistic (e.g. *F*, *t*, *r*) with confidence intervals, effect sizes, degrees of freedom and *P* value noted<br>*Give P values as exact values whenever suitable.*
☒	☐	For Bayesian analysis, information on the choice of priors and Markov chain Monte Carlo settings
☒	☐	For hierarchical and complex designs, identification of the appropriate level for tests and full reporting of outcomes
☒	☐	Estimates of effect sizes (e.g. Cohen's *d*, Pearson's *r*), indicating how they were calculated

Our web collection on statistics for biologists contains articles on many of the points above.

Software and code

Policy information about availability of computer code

Data collection	Imaging: ZEISS LSM 800 (confocal imaging) and ZEISS AXIO Zoom.V16 (WISH imaging).<br>Sequencing data: Illumina NovaSeq 6000.<br>Luciferase data: BioTek Synergy HTX.<br>Flow cytometry data: Beckman Coulter.
Data analysis	ScRNA-Seq analysis: CellRanger (6.0.1), Seurat (3.2.3), Monocle (0.2.3.0), and velocyto.R (0.6).<br>Bulk RNA-Seq: STAR (2.7.10b), featureCounts (Rsubread, 2.0.1), and DESeq2 (1.26.0).<br>ChIP-Seq: BWA-MEM (0.7.17), Samtools (1.15.1), MACS2 (2.2.7.1), ChIPseeker (1.22.1), and deepTools (3.5.1).<br>Statistical analysis: GraphPad Prism (8.0.2).

For manuscripts utilizing custom algorithms or software that are central to the research but not yet described in published literature, software must be made available to editors and reviewers. We strongly encourage code deposition in a community repository (e.g. GitHub). See the Nature Portfolio guidelines for submitting code & software for further information.

Data

Policy information about availability of data

All manuscripts must include a data availability statement. This statement should provide the following information, where applicable:

- Accession codes, unique identifiers, or web links for publicly available datasets
- A description of any restrictions on data availability
- For clinical datasets or third party data, please ensure that the statement adheres to our policy

Our sequencing data were all uploaded to the GEO Repository (GSE198314, https://www.ncbi.nlm.nih.gov/geo/query/acc.cgi?acc=GSE198314). The zebrafish genome version used in the present study is GRCz11.

Research involving human participants, their data, or biological material

Policy information about studies with human participants or human data. See also policy information about sex, gender (identity/presentation), and sexual orientation and race, ethnicity and racism.

Reporting on sex and gender	N/A
Reporting on race, ethnicity, or other socially relevant groupings	N/A
Population characteristics	N/A
Recruitment	N/A
Ethics oversight	N/A

Note that full information on the approval of the study protocol must also be provided in the manuscript.

Field-specific reporting

Please select the one below that is the best fit for your research. If you are not sure, read the appropriate sections before making your selection.

☒ Life sciences ☐ Behavioural & social sciences ☐ Ecological, evolutionary & environmental sciences

For a reference copy of the document with all sections, see nature.com/documents/nr-reporting-summary-flat.pdf

Life sciences study design

All studies must disclose on these points even when the disclosure is negative.

Sample size	No methods was used to predetermine the sample sizes. The sample sizes were chosen based on previous reports (10.3324/haematol.2018.194316; 10.1182/blood-2015-12-686147; 10.1182/blood-2011-03-342501) and were clearly indicated in the figure legends.
Data exclusions	No data was excluded.
Replication	All experiments were repeated for 2-3 times, which were clearly indicated in the figure legends.
Randomization	The allocations of zebrafish and mice were random.
Blinding	In WISH assays, we are blinded to the genotypes of larvae when counting the eosinophil number of each fish. Heterozygous adult fish were intercrossed to generate wild type and mutant larvae, which were firstly applied to WISH as a pool to detect their eosinophil population. Afterwards, the larvae were genotyped and the relevant data were grouped. For other experiments, blinding was not needed because the results were quantitive and did not require subjective judgement.

Behavioural & social sciences study design

All studies must disclose on these points even when the disclosure is negative.

Study description	
Research sample	
Sampling strategy	

Data collection

Timing

Data exclusions

Non-participation

Randomization

Ecological, evolutionary & environmental sciences study design

All studies must disclose on these points even when the disclosure is negative.

Study description

Research sample

Sampling strategy

Data collection

Timing and spatial scale

Data exclusions

Reproducibility

Randomization

Blinding

Did the study involve field work? ☐ Yes ☐ No

Field work, collection and transport

Field conditions

Location

Access & import/export

Disturbance

Reporting for specific materials, systems and methods

We require information from authors about some types of materials, experimental systems and methods used in many studies. Here, indicate whether each material, system or method listed is relevant to your study. If you are not sure if a list item applies to your research, read the appropriate section before selecting a response.

Materials & experimental systems

n/a	Involved in the study	
☐	☒	Antibodies
☐	☒	Eukaryotic cell lines
☒	☐	Palaeontology and archaeology
☐	☒	Animals and other organisms
☒	☐	Clinical data
☒	☐	Dual use research of concern
☒	☐	Plants

Methods

n/a	Involved in the study	
☐	☒	ChIP-seq
☐	☒	Flow cytometry
☒	☐	MRI-based neuroimaging

Antibodies

Antibodies used	The following antibodies were used: GFP (Abcam, ab6658, 1:200), CD34 (RAM34, ThermoFisher, 13-0341-81, 1:100), CD11b (M1/70, ThermoFisher, 63-0112-80, 1:400), CD16/32 (2.4G2, BD Horizon, 565502, 1:400), CD115 (AFS598, Biolegend, 135510, 1:400), cKit (2B8, ThermoFisher, 62-1171-82, 1:400), CD101 (BB27, Biolegend, 331007, 1:400), CXCR4 (2B11, ThermoFisher, 13-9991-82, 1:400), Gr1 (RB6-8C5, ThermoFisher, 45-5931-80, 1:800), Ly6C (HK1.4, Biolegend, 128026, 1:400), SiglecF (E50-2440, BD Biosciences, 562757, 1:400), CD106 (429, Biolegend, 105716, 1:400), Ly6G (1A8, Biolegend, 127618, 1:200), CD90.2 (53-2.1, Biolegend, 140314, 1:400), B220 (RA3-6B2, ThermoFisher, 13-0452-82, 1:400), and NK1.1 (PK136, ThermoFisher, 13-5941-81, 1:400).
Validation	The anti-GFP antibody was used for ChIP-Seq, which was validated in our previous publication (Mei wu et al., Front Cell Dev Bio, 2021), and all other antibodies were used for the flow cytometry of mouse bone marrow cells, which was as reported before (Immanuel Kwok et al., Immunity, 2020).

Eukaryotic cell lines

Policy information about cell lines and Sex and Gender in Research

Cell line source(s)	The only cell line used in our study is the HEK-293T cell line, obtained from ATCC.
Authentication	The HEK-293T cell line was obtained from ATCC and authenticated by China Center for Type Culture Collection (CCTCC) through STR profiling.
Mycoplasma contamination	No evidence of mycoplasma contamination was observed using mycoplasma detection kit (Vazyme, China).
Commonly misidentified lines (See ICLAC register)	No commonly misidentified line was used in the study.

Palaeontology and Archaeology

Specimen provenance	
Specimen deposition	
Dating methods	

☐ Tick this box to confirm that the raw and calibrated dates are available in the paper or in Supplementary Information.

Ethics oversight	

Note that full information on the approval of the study protocol must also be provided in the manuscript.

Animals and other research organisms

Policy information about studies involving animals; ARRIVE guidelines recommended for reporting animal research, and Sex and Gender in Research

Laboratory animals	The zebrafish used in this study were all based on AB strain. The zebrafish lines used were: wild type, Tg(gata2a:eGFP), Tg(cdh17:DsRed), Tg(lyz:DsRed), Tg(mpeg1:loxP-DsRed-loxP-eGFP), Tg(coro1a:loxP-DsRed-loxP-eGFP), cebp1smu1 mutant, cebpbszy7 mutant, and our generated Tg(hsp70:cebp1-eGFP), Tg(hsp70:cebpb-eGFP), Tg(eslec:eGFP) and Tg(eslec:DsRed) lines. Larval zebrafish of all above fish were used, while adult fish of wild type, cebp1 mutant, cebpb mutant, and Tg(eslec:eGFP) were also used.<br>For mouse, six- to ten-week s old Mrp8creCebpefl/fl and Cebpefl/fl mice were used for flow cytometry. The mice were bred and maintained under SPF conditions on a 12h light/12h darkness schedule at 22.5 °C, 52.5% humidity.
Wild animals	This research does not involve wild animals.
Reporting on sex	Sex was considered in some of our study design. Larval and juvenile zebrafish: unable to distinguish the gender. Adult zebrafish and mice: sex not distinguished, except for the quantitative scRNA-Seq analysis of wild type and mutant zebrafish KM cells.
Field-collected samples	This research does not involve field-collected sample.
Ethics oversight	All studies involving zebrafish are reviewed by the Animal Research Advisory Committee of the South China University of Technology. All studies involving mice were performed under the approval of the Institutional Animal Care and Use Committee, in accordance with the guidelines of the Agri-Food and Veterinary Authority and the National Advisory Committee for Laboratory Animal Research of Singapore.

Note that full information on the approval of the study protocol must also be provided in the manuscript.

Clinical data

Policy information about clinical studies

All manuscripts should comply with the ICMJE guidelines for publication of clinical research and a completed CONSORT checklist must be included with all submissions.

Clinical trial registration	
Study protocol	
Data collection	
Outcomes	

Dual use research of concern

Policy information about dual use research of concern

Hazards

Could the accidental, deliberate or reckless misuse of agents or technologies generated in the work, or the application of information presented in the manuscript, pose a threat to:

No	Yes	
☐	☐	Public health
☐	☐	National security
☐	☐	Crops and/or livestock
☐	☐	Ecosystems
☐	☐	Any other significant area

Experiments of concern

Does the work involve any of these experiments of concern:

No	Yes	
☐	☐	Demonstrate how to render a vaccine ineffective
☐	☐	Confer resistance to therapeutically useful antibiotics or antiviral agents
☐	☐	Enhance the virulence of a pathogen or render a nonpathogen virulent
☐	☐	Increase transmissibility of a pathogen
☐	☐	Alter the host range of a pathogen
☐	☐	Enable evasion of diagnostic/detection modalities
☐	☐	Enable the weaponization of a biological agent or toxin
☐	☐	Any other potentially harmful combination of experiments and agents

Plants

Seed stocks	N/A
Novel plant genotypes	N/A
Authentication	N/A

ChIP-seq

Data deposition

☒ Confirm that both raw and final processed data have been deposited in a public database such as GEO.

☒ Confirm that you have deposited or provided access to graph files (e.g. BED files) for the called peaks.

Data access links<br>*May remain private before publication.*	The ChIP-Seq data were uploaded to the GEO Repository (GSE198310).
Files in database submission	In our GEO dataset, the raw data and processed data (Bigwig files) for all the 8 samples were uploaded. For the ChIP-Seq assay to reveal Cebp1-binding genes, "cebp1_ChIP_1" and "cebp1_ChIP_2" were samples with GFP antibody precipitation, while "cebp1_input_1" and "cebp1_input_2" served as input controls. For the ChIP-Seq assay to reveal Cebpβ-binding genes, "cebpb_ChIP_1" and "cebpb_ChIP_2" were samples with GFP antibody precipitation, while "cebpb_input_1" and "cebpb_input_2" served as input controls.
Genome browser session<br>(e.g. UCSC)	We used Integrative Genomics Viewer to visualize the peaks from Bigwig files.

Methodology

Replicates	Two replicates were applied for each group. Each replicate was obtained from different indiviuduals (~200 larvae as a pool for each replicate).
Sequencing depth	Pair-end reads were sequenced for each sample with 6 G raw data.
Antibodies	The antibody used in this study is anti-GFP antibody (Abcam goat anti-GFP, ab6658).
Peak calling parameters	Peak calling was performed with macs2 (2.2.7.1) with default parameters (-B -q 0.05).
Data quality	In the Cebp1 ChIP-Seq data, 586 peaks had a q value lower than 0.05, and 96 peaks had a signalValue higher than 5. In the Cebpβ ChIP-Seq data, 3,796 peaks had a q value lower than 0.05, and 661 peaks had a signalValue higher than 5.
Software	Mapping was performed using BWA-MEM (0.7.17). Aligned reads were filtered with Samtools (1.15.1) and peak calling was performed with macs2 (2.2.7.1). The peaks were annotated with ChIPseeker (1.22.1). Bigwig files were generated with deepTools (3.5.1).

Flow Cytometry

Plots

Confirm that:

☒ The axis labels state the marker and fluorochrome used (e.g. CD4-FITC).

☒ The axis scales are clearly visible. Include numbers along axes only for bottom left plot of group (a 'group' is an analysis of identical markers).

☒ All plots are contour plots with outliers or pseudocolor plots.

☒ A numerical value for number of cells or percentage (with statistics) is provided.

Methodology

Sample preparation	Larval and juvenile zebrafish were ground and digested with Dispase. For adult fish, kidney was dissected and pipitte-mix into cell suspensions. Intraperinoteal exudate and perpheral blood were collected and directly applied to flow cytometry. For spleen, intestine, brain, liver, and heart, organs were digested with Collagenase IV into cell suspensions.
Instrument	Beckman Coulter
Software	FlowJo
Cell population abundance	For each sample, 1,000,000 total cells were input (except for those don't have enough cells in the whole tissue). After gating, eosinophils occupy ~0-8% of the total cells, depending on the genotypes and tissue types.
Gating strategy	All samples were firstly gated by preliminary FSC/SSC gate to exclude cell fragments and cell clumps. Then, since all our samples were collected from Tg(eslec:eGFP) fish, in which eosinophils were labeled with eGFP, we gated the eosinophils on eGFP+DsRed- strategy to exclude autoflorescent cells.

☒ Tick this box to confirm that a figure exemplifying the gating strategy is provided in the Supplementary Information.

Magnetic resonance imaging

Experimental design

Design type

Design specifications

Behavioral performance measures

Acquisition

Imaging type(s)

Field strength

Sequence & imaging parameters

Area of acquisition

Diffusion MRI ☐ Used ☐ Not used

Preprocessing

Preprocessing software

Normalization

Normalization template

Noise and artifact removal

Volume censoring

Statistical modeling & inference

Model type and settings

Effect(s) tested

Specify type of analysis: ☐ Whole brain ☐ ROI-based ☐ Both

Statistic type for inference

(See Eklund et al. 2016)

Correction

Models & analysis

n/a	Involved in the study
☐	☐ Functional and/or effective connectivity
☐	☐ Graph analysis
☐	☐ Multivariate modeling or predictive analysis

Functional and/or effective connectivity

Graph analysis

Multivariate modeling and predictive analysis
